# Performance and Fiber-Induced Modification Mechanisms of Geopolymer Recycled Aggregate Porous Concrete: Effects of Fiber Type and Content

**DOI:** 10.3390/ma19081544

**Published:** 2026-04-13

**Authors:** Xinyu Bai, Yu Luo, Gang Zheng, Yu Diao, Peishu Huo, Zheng Che, Xiaomin Liu, Yun Zhao

**Affiliations:** 1 School of Civil Engineering, Tianjin University, Tianjin 300072, China; tjubxy@163.com (X.B.);; 2 China Construction Sixth Engineering Bureau Co., Ltd., Tianjin 300450, Chinachezheng0314@163.com (Z.C.);; 3 State Key Laboratory of Hydraulic Engineering Intelligent Construction and Operation, Tianjin University, Tianjin 300072, China; 4 Key Laboratory of Coastal Civil Engineering Structure and Safety, Ministry of Education, Tianjin 300350, China; 5 China Construction Sixth Engineering Bureau Hydropower Construction Co., Ltd., Tianjin 300222, China

**Keywords:** geopolymer, coir fiber, basalt fiber, steel fiber, recycled aggregate, porous concrete, effective porosity, compressive strength, SEM analysis

## Abstract

**Highlights:**

- First proposed geopolymer recycled aggregate porous concrete (GRAPC) for low-carbon construction.
- Optimized GRAPC mix: 40% slag, alkali modulus 1.4, alkali content 8%.
- 0.25 vol% steel fibers raised compressive strength by about 25% and porosity by 17.4%.
- Fibers transformed the failure mode from brittle to ductile, with coir fibers best for toughness.
- SEM revealed coir/steel fibers dispersed well, while basalt fibers agglomerated.
- Allows production of GRAPC with tailored properties for specific engineering needs.
- Shows great potential to cut carbon emissions and utilize solid waste in construction.
- Guides the design of ductile, porous, and eco-friendly GRAPC through targeted fiber selection.

**Abstract:**

Environmental concerns associated with the construction industry have drawn increasing attention worldwide. This study addresses the dual challenges of carbon emissions from cement production and construction waste disposal by developing and characterizing a fiber-modified geopolymer recycled aggregate porous concrete (GRAPC). An orthogonal experiment first optimized the GRAPC mix proportion (slag content = 40%, alkali modulus = 1.4, alkali content = 8%). Subsequently, the effects of coir, basalt, and steel fibers (0.25% and 0.5%) on its properties were investigated through laboratory experiments combined with scanning electron microscopy (SEM) analysis. The results show that steel fibers at 0.25% dosage enhanced compressive strength by approximately 25% due to their effective stress-bearing capacity. In contrast, 0.5% coir and basalt fibers reduced compressive strength by approximately 20.5% and 22.2%, respectively, due to low intrinsic strength and agglomeration. In addition, 0.25% coir and steel fibers increased effective porosity by 18.4% and 17.4%, respectively, owing to their uniform dispersion. All fibers promoted a more ductile-like failure mode, with coir fibers providing the best toughness improvement. This study elucidates how fiber type and dosage regulate the macro-properties and micro-mechanisms of GRAPC, providing a basis for designing sustainable eco-friendly concrete with great potential for non-primary load-bearing engineering fields.

## 1. Introduction

The construction industry is a major contributor to global carbon emissions and energy consumption. Portland cement production consumes approximately 1.5 metric tons of raw materials and emits about 0.8 metric tons of CO_2_ per ton of cement [1], in addition to requiring over 2.72 GJ of thermal energy and 65 kWh of electrical energy per ton [2,3]. Alkali-activated geopolymer materials offer a more environmentally friendly alternative, with reduced CO_2_ emissions compared to Portland cement [4]. Meanwhile, the construction sector generates massive amounts of demolition waste, with over 100 million metric tons produced annually in the U.S. [5], posing significant environmental challenges. To mitigate the environmental impact of construction waste disposal, recycled aggregates have been adopted in concrete production [6,7]. The combination of geopolymer binder and recycled aggregate presents a promising sustainable solution.

Geopolymer recycled aggregate concrete has attracted considerable research attention. Hadi et al. [8] investigated the water-to-binder ratio and slag content in geopolymers. The results indicated that as the water-to-binder ratio decreases and the slag content increases, the reaction rate of the geopolymer accelerates, and the strength of the resulting product is enhanced. The study by Görhan et al. [9] demonstrated that as the concentration of the alkali activator increases, the compressive strength of concrete first rises and then declines. Elevated temperature curing, however, can promote a more complete geopolymerization reaction, thereby enhancing the mechanical performance of the concrete. Several researchers have investigated the differences in mechanical properties between geopolymer recycled aggregate concrete and conventional recycled aggregate concrete [10,11,12,13]. The results show that the compressive strength of concrete decreases as the replacement ratio of recycled aggregates increases. However, the alkaline environment of the geopolymer can activate the old mortar attached to the surface of the recycled aggregates, thereby enhancing the strength of the interfacial transition zone between the recycled aggregates and the geopolymer matrix.

Despite its environmental benefits, the pronounced brittleness of geopolymer recycled aggregate concrete limits its engineering application [14,15]. To address this issue of high brittleness and low ductility, numerous scholars have incorporated fibers as reinforcement and have conducted relevant research in this area. Sukontasukkul et al. [16] investigated the influence of fiber type on geopolymer concrete. The study revealed that steel fibers provide superior improvement in the flexural toughness of concrete compared to polypropylene fibers. Bhutta et al. [17] studied the effects of different fiber types and contents on the mechanical properties of geopolymer concrete. The results indicated that incorporating steel fibers enhances the flexural strength of concrete, with hooked-end steel fibers demonstrating the most effective modification for geopolymer concrete. Nevertheless, the inherent brittleness of geopolymer materials and the initial defects in recycled aggregates continue to limit the applicability of geopolymer recycled aggregate concrete for primary load-bearing components in building and structural engineering.

While fiber-reinforced geopolymer recycled aggregate concrete has been studied in dense forms [18,19], the combination of geopolymer binder, recycled aggregate, and porous concrete architecture with fiber reinforcement remains unexplored. Compared with traditional concrete, porous concrete exhibits excellent characteristics in terms of drainage, water purification, and sound absorption [20,21,22,23,24], giving it broad application prospects in non-primary load-bearing engineering fields such as slope protection, road paving, and municipal landscaping. If geopolymer and recycled aggregates are used as raw materials, combined with the addition of fibers to prepare porous concrete, the defects of relatively low strength and pronounced brittleness can be overcome, endowing the material with both environmental friendliness and practical applicability.

To address this gap, this study first employed a three-factor, three-level orthogonal experimental design to determine the optimal mix proportion for geopolymer recycled aggregate porous concrete (GRAPC). Subsequently, fiber-reinforced porous concrete was fabricated by incorporating three types of fibers—coir, basalt, and steel—at varying dosages (0%, 0.25%, and 0.5%, respectively). The compressive strength and porosity of the specimens were measured through laboratory tests, and scanning electron microscopy (SEM) was utilized to observe their mesoporous structure and fiber distribution characteristics, aiming to elucidate the influence of fiber type and content on the physical and mechanical properties of the concrete.

## 2. Materials and Methods

### 2.1. Materials and Mix Proportion Design

#### 2.1.1. Raw Materials

The raw materials used in this experiment were Grade S95 ground granulated blast furnace slag (GGBS, Gongyi Longze Water Purification Material Co., Ltd., Gongyi, Henan, China) and Grade I fly ash (FA, Shijiazhuang Shangan Power Plant, Shijiazhuang, Hebei, China). The specific surface area of the S95 GGBS was 429 m^2^/kg, with a density of 3.1 g/cm^3^, while the Grade I FA had a density of 2.6 g/cm^3^. The chemical compositions of the GGBS and FA are presented in Table 1. The alkali activator solution was prepared from sodium silicate powder (modulus of 2.8, Henan Borun Casting Materials Co., Ltd., Zhengzhou, Henan, China) and flake sodium hydroxide solid (purity > 99%, Dongguan Yangtuo Automation Technology Co., Ltd., Dongguan, Guangdong, China). A single-sized recycled coarse aggregate with a particle size of 10~20 mm was selected (Zhengzhou Yuanshifa Environmental Protection Technology Co., Ltd., Zhengzhou, Henan, China). In addition, due to the presence of various impurities in the recycled aggregate, it was necessary to wash the aggregate to avoid affecting the bond between the aggregate and the paste matrix. The washing procedure consisted of manually removing visible impurities, followed by rinsing with a high-pressure water gun. After washing, the aggregate was air-dried in a well-ventilated area until reaching a stable moisture condition before mixing. The fundamental properties of the recycled aggregate are listed in Table 2. Both the coir fibers and basalt fibers were cut to a length of approximately 3 cm, with densities of 1.22 g/cm^3^ and 2.69 g/cm^3^, respectively. The steel fibers employed were galvanized straight fibers with a length of 2.5 cm and a diameter of 0.5 mm, exhibiting a density of 7.5 g/cm^3^. Considering the aggregate size of 10~20 mm, the more flexible coir and basalt fibers were cut to 3 cm to ensure effective bridging between aggregates, while the stiffer steel fibers were cut to 2.5 cm to avoid mixing constraints.

#### 2.1.2. Optimization of GRAPC Baseline Mix Proportion via Orthogonal Experiment

An orthogonal experimental design was employed to determine the optimal mix proportion for GRAPC. The 28-day cube compressive strength of the concrete was selected as the response index. The effects of three factors were investigated: slag content, alkali activator modulus, and alkali content. The levels of each factor were selected based on the literature recommendations [25,26,27]: the slag content was set at 40%, 60%, and 80%; the alkali activator modulus was set at 1.2, 1.4, and 1.6; and the alkali content was set at 8%, 10%, and 12%. Through preliminary tests, the aggregate-to-binder ratio and water-to-binder ratio were held constant at 4.5 and 0.38, respectively, to ensure that the aggregates were adequately coated by the binder while avoiding visible paste sedimentation. The mix proportions of the orthogonal test are presented in Table 3.

#### 2.1.3. Design of Fiber-Reinforced GRAPC Mixes

To study the effect of fibers, all specimens were prepared using the optimized mix proportion: an aggregate-to-binder ratio of 4.5, water-to-binder ratio of 0.38, slag content of 40%, alkali activator modulus of 1.4, and alkali content of 8%. Guided by preliminary tests, the fiber volume fractions were set at 0.25% and 0.5% to ensure adequate workability of the fresh concrete and the integrity of the cast specimens. The effective porosity and 7-day cube compressive strength were selected as the key indicators to evaluate the effect of fibers on the physical and mechanical properties of GRAPC. A 7-day curing age was adopted for the fiber-reinforced tests to enable rapid screening of fiber type and dosage, while all specimens in this series were subjected to the same curing conditions to ensure internal consistency. The detailed mix proportions are presented in Table 4.

### 2.2. Specimen Preparation

**Figure 1:**
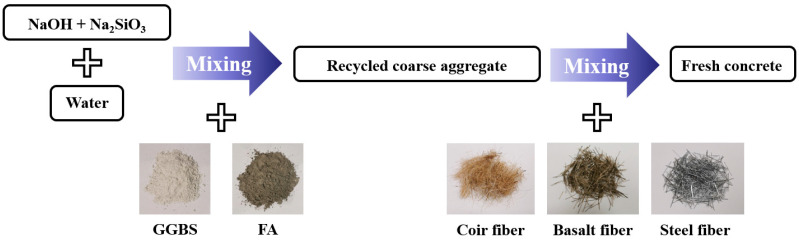
Flow chart of the specimen preparation process for fiber-reinforced geopolymer recycled aggregate porous concrete.

The alkali activator solution was prepared by dissolving NaOH and Na_2_SiO_3_ and then slowly pouring it into a pre-mixed blend of slag and fly ash to form a geopolymer paste. Subsequently, the thoroughly mixed paste was gradually added to the pre-weighed recycled aggregates and blended homogeneously to ensure uniform coating of the aggregate surfaces with the paste. Fibers were then introduced into the mixture in three batches, with thorough mixing after each addition. Following mixing, the concrete was placed into cubic molds in two layers and compacted manually using a tamping rod. Each layer was filled to approximately equal depth and compacted with 29 uniformly distributed strokes in a spiral motion from the perimeter towards the center. All specimens were demolded 24 h after casting. After demolding, the specimens were transferred to a standard curing chamber for 7 days before subsequent testing. The preparation process is illustrated in Figure 1.

### 2.3. Testing and Characterization Methods

#### 2.3.1. Effective Porosity

The effective porosity of the specimens was measured using the water displacement method [28]. For each mix proportion, three specimens were tested, and the average value was used as the effective porosity for that mix. First, the specimens were placed in an oven at (105 ± 5) °C and dried to a constant weight. After cooling to room temperature, the volume (*V*) and dry weight (*W*_d_) of each specimen were determined. Subsequently, the specimens were immersed in water to ensure that all open pores were fully saturated. After no more air bubbles emerged, the submerged weight (*W*_w_) was recorded. Finally, the effective porosity of the porous concrete was calculated using the following formula:

(1) $$P=(1-\frac{W_{d}-W_{w}}{\rho_{w}V})\times 100\%,$$

where *P* is the effective porosity; *W*_d_ is the dry weight of the specimen in air (kg); *W*_w_ is the submerged weight of the specimen in water (kg); *V* is the volume of the specimen (m^3^); and *ρ*_w_ is the density of water (kg/m^3^).

#### 2.3.2. Compressive Strength

Compressive strength tests were conducted on three 100 mm × 100 mm × 100 mm cube specimens for each mix proportion using a YAW-1000 microcomputer-controlled electro-hydraulic servo pressure testing machine (Changchun Kexin Test Instruments Co., Ltd., Changchun, Jilin, China), with a loading rate of 0.5 MPa/s. The average value of the compressive strengths of three specimens in each group, multiplied by a size conversion factor of 0.95, was recorded as the cube compressive strength for that group of concrete specimens.

#### 2.3.3. Microstructural Characterization (SEM)

After the uniaxial compression tests, fragments with fracture surfaces were collected for microstructural examination. A ZEISS GeminiSEM 300 (Carl Zeiss AG, Oberkochen, Germany) scanning electron microscope was employed to observe and analyze the microstructure of the paste matrix, aggregates, and fibers at the fracture interfaces.

## 3. Results

### 3.1. Optimization of the GRAPC Mix Proportion

#### 3.1.1. Effect of Slag Content

**Figure 2:**
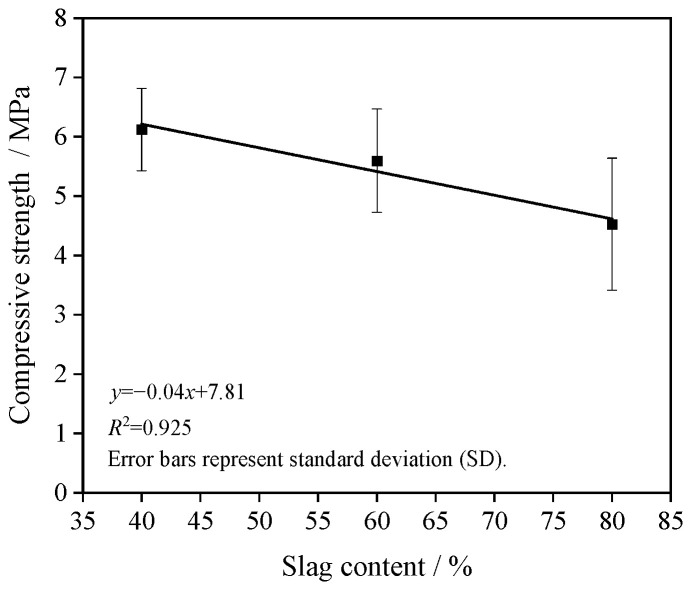
Effect of slag content on compressive strength.

Figure 2 illustrates the variation in compressive strength of GRAPC with respect to the slag content. The compressive strength exhibits an approximately linear decreasing trend as the slag content increases. When the slag content rises from 40% to 80%, the compressive strength declines from 6.1 MPa to 4.5 MPa, representing a reduction of approximately 26.2%.

#### 3.1.2. Effect of Alkali Activator Modulus

**Figure 3:**
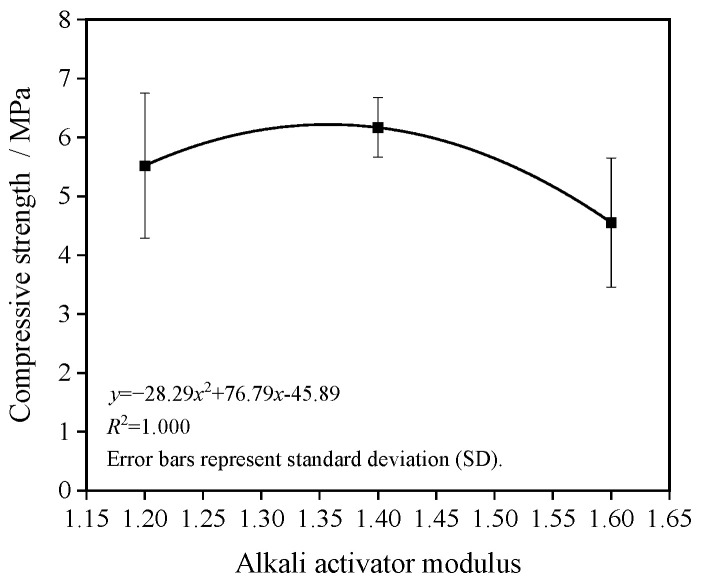
Effect of alkali activator modulus on compressive strength.

Figure 3 illustrates the effect of alkali activator modulus on the compressive strength of the porous concrete. The compressive strength initially increases and then decreases with increasing modulus. When the modulus increases from 1.2 to 1.4, the compressive strength rises from 5.5 MPa to 6.2 MPa, representing an increase of 12.7%. However, as the modulus further increases to 1.6, the strength declines to 4.6 MPa, a decrease of 25.8%.

#### 3.1.3. Effect of Alkali Content

**Figure 4:**
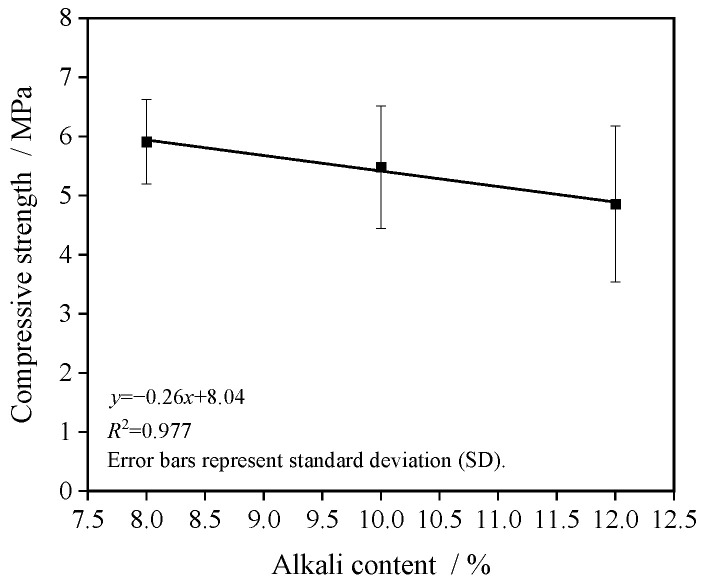
Effect of alkali content on compressive strength.

Figure 4 illustrates the influence of alkali content on the compressive strength of the porous concrete. The compressive strength exhibits an approximately linear decreasing trend with increasing alkali content. Specifically, as the alkali content rises from 8% to 12%, the compressive strength progressively declines from 5.9 MPa to 4.9 MPa.

Therefore, the optimal mix proportion for the baseline GRAPC was determined as follows: aggregate-to-binder ratio = 4.5, water-to-binder ratio = 0.38, slag content = 40%, alkali activator modulus = 1.4, and alkali content = 8%.

### 3.2. Effective Porosity of Fiber-Reinforced GRAPC

**Figure 5:**
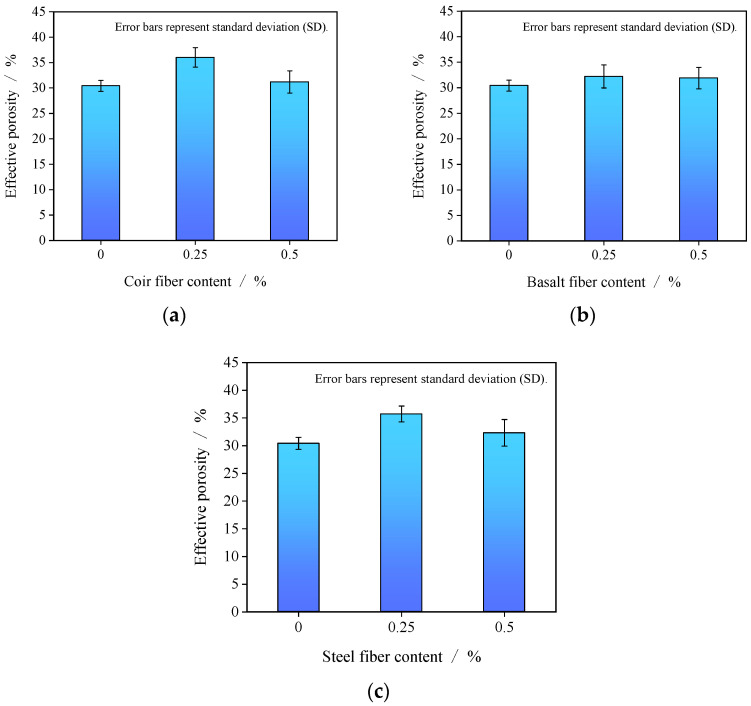
Effective porosity of porous concrete. (**a**) Coir fiber; (**b**) basalt fiber; (**c**) steel fiber.

Figure 5 presents the measured effective porosity of the porous concrete with different fiber contents. For all fiber types, the effective porosity initially increased and then decreased as the fiber content rose. With coir and steel fibers, when the fiber content increased from 0% to 0.25%, the effective porosity rose from 30.4% to 36.0% and 35.7%, respectively. As the fiber content further increased from 0.25% to 0.5%, the effective porosity decreased to 31.2% and 32.3%, respectively. For basalt fibers, the effective porosity was 30.4 ± 1.1% in the control group, increased to 32.2 ± 2.3% at 0.25% content, and was 31.9 ± 2.1% at 0.5% content. Considering the experimental variability, the difference between the 0.25% and 0.5% basalt fiber groups falls within the range of standard deviation, indicating that increasing the basalt fiber dosage from 0.25% to 0.5% had no notable effect on porosity.

### 3.3. Compressive Strength of Fiber-Reinforced GRAPC

**Figure 6:**
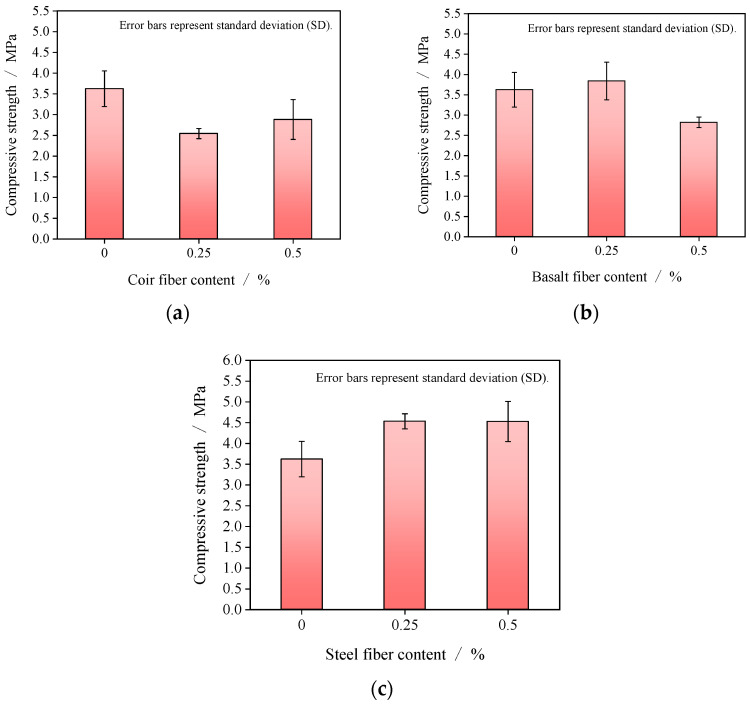
Compressive strength of porous concrete. (**a**) Coir fiber; (**b**) basalt fiber; (**c**) steel fiber.

Figure 6 illustrates the variation in compressive strength of GRAPC with fiber content. The compressive strength of the porous concrete first decreased and then increased with increasing coir fiber content, as shown in Figure 6a. When the coir fiber content increased from 0% to 0.5%, the compressive strength decreased by approximately 20.5%. The compressive strength initially rose and then declined with increasing basalt fiber content, as shown in Figure 6b. An increase in basalt fiber content from 0% to 0.25% led to a 6% increase in compressive strength. However, a further increase from 0.25% to 0.5% resulted in a reduction in compressive strength of approximately 26.6%. Under the tested conditions, both coir and basalt fibers resulted in a net decrease in the compressive strength of GRAPC. For steel fibers, the compressive strength initially increased and then plateaued, as shown in Figure 6c. The strength of GRAPC increased from 3.6 MPa to 4.5 MPa when the fiber content rose from 0% to 0.25%, and remained at 4.5 MPa with a further increase to 0.5% content.

### 3.4. Microstructural Analysis of Fiber-Reinforced GRAPC

**Figure 7:**
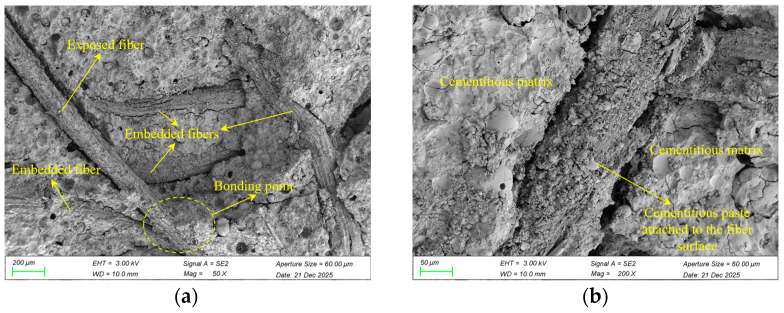

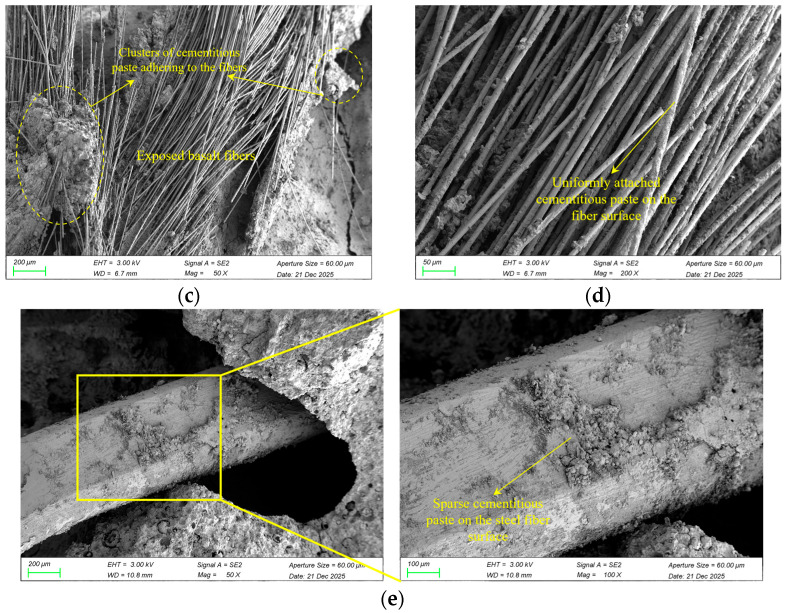
Representative scanning electron microscopy (SEM) images of the three fiber types in geopolymer recycled aggregate porous concrete (GRAPC). (**a**) Coir fiber at 50× magnification; (**b**) coir fiber at 200× magnification; (**c**) basalt fibers at 50× magnification; (**d**) basalt fibers at 200× magnification; (**e**) steel fiber at 50× magnification and 100× magnification.

Figure 7a,b show the microstructure of coir fibers within the GRAPC. The coir fibers are separated from each other and independently distributed within the concrete. Some fibers appear partially embedded in the matrix, and their surfaces are coated with a relatively uniform layer of geopolymer material, as shown in Figure 7b. Figure 7c,d present the SEM results of basalt fibers within the concrete. Figure 7c (at 50× magnification) shows that the fine-diameter fibers form bundles of varying thicknesses. Figure 7d (at 200× magnification) reveals that for the vast majority of individual basalt fibers, their surfaces are uniformly coated with geopolymer paste. Figure 7e shows the microstructure of steel fibers within the concrete. At 50× magnification (left), the fibers exhibit a large diameter. At higher magnification (right), the interface detail is shown, where only a sparse and uneven layer of geopolymer paste coats the fiber surface.

## 4. Discussion

### 4.1. Analysis of Failure Modes: From Brittle Fracture to Ductile Integrity

#### 4.1.1. Brittle Fracture of Plain GRAPC

**Figure 8:**
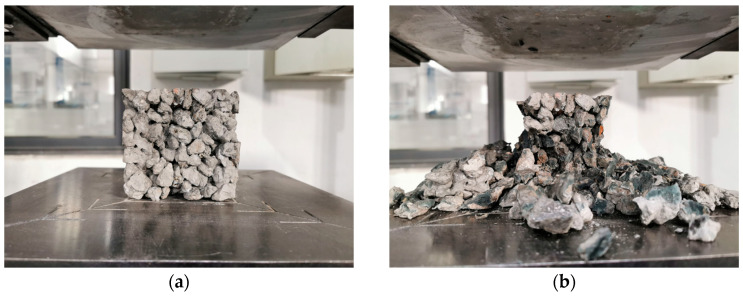
Compressive failure of concrete without fibers. (**a**) Before failure; (**b**) after failure.

The compressive failure of plain GRAPC exhibited a classic brittle fracture mode, as detailed in the following observed sequence and shown in Figure 8. It was found that shortly after loading commenced, localized aggregate spalling first occurred at the upper and lower corners of the specimen. Subsequently, with continued loading, visible cracks appeared between the aggregates. As the load further increased, the number of cracks grew, and they gradually propagated. When adjacent cracks interconnected, merged with each other, and formed failure surfaces together with the concrete pores, the specimen failed completely. This was followed by extensive aggregate collapse, and the loading was terminated. Ultimately, the penetrating failure surfaces were roughly vertically oriented and located near the vertical side surfaces of the specimen. Consequently, the plain GRAPC lacks the toughness required for many structural applications, which fundamentally motivates the incorporation of fibers to alter this failure mechanism, similar to the brittle failure behavior reported for geopolymer concrete [29].

#### 4.1.2. Transition to Ductile Integrity in Fiber-Reinforced GRAPC

**Figure 9:**
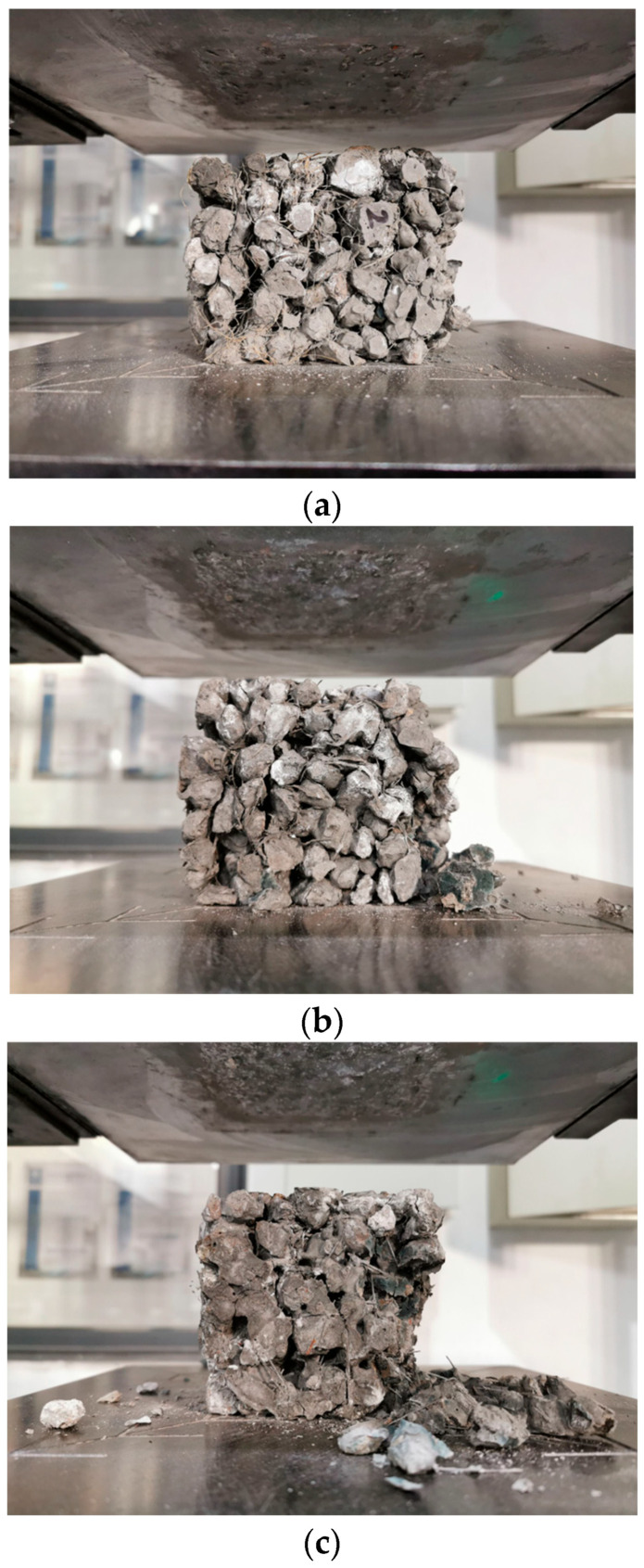
Compressive failure of concrete with fibers. (**a**) Coir fiber; (**b**) basalt fibers; (**c**) steel fibers.

In stark contrast to the plain GRAPC, the incorporation of fibers fundamentally altered the failure mode, as shown in Figure 9. This transition is governed by the distinct fiber–matrix interactions and dispersion states.

Coir fibers yielded the most pronounced improvement in ductility, as shown in Figure 9a. The well-dispersed fibers formed a cohesive network within the matrix, as shown in Figure 7a,b. Microscopic observations reveal that individual coir fibers are either embedded within the geopolymer matrix or bonded at their ends, with mid-sections bridging across pores. This unique configuration allows them to interlock and create a relatively complete three-dimensional network. Upon cracking, this continuous network provides effective bridging across cracks, restraining aggregate displacement and preventing spalling, thereby resulting in the characteristic “damaged yet integral” morphology, which is consistent with the interfacial toughening mechanism reported for fiber-reinforced geopolymer composites [30]. In contrast, basalt fibers also mitigated brittleness, but to a lesser extent, as revealed in Figure 9b. While they improved integrity compared to the plain concrete, their tendency to agglomerate limited the formation of a uniform, effective bridging network. Regarding steel fibers, Figure 9c shows that after failure, some aggregates spalled along the failure surfaces, and the specimen exhibited relatively obvious brittleness. This is primarily because steel fibers have significantly lower hydrophilicity compared to coir and basalt fibers. Additionally, the high stiffness of steel fibers prevents them from effectively coating the aggregate surfaces together with the geopolymer paste to form a fibrous network, which in turn fails to adequately restrain and bridge the aggregates after specimen failure.

Overall, the enhancement of ductility depends on whether fibers can form a uniformly dispersed three-dimensional network within the matrix, thereby effectively restraining aggregate spalling during compression. Coir fibers, owing to their excellent dispersion and strong bonding with the geopolymer matrix, achieve the most significant transition from brittle fracture to ductile integrity.

### 4.2. Mechanisms of Fiber-Induced Modifications on Macro-Properties

#### 4.2.1. Mechanisms of Fiber Type and Content in Porosity Regulation

For all fiber types, the effective porosity initially increased and then decreased as the fiber content rose, as shown in Figure 5, indicating that incorporating an appropriate amount of fibers into GRAPC can effectively increase its effective porosity.

**Figure 10:**
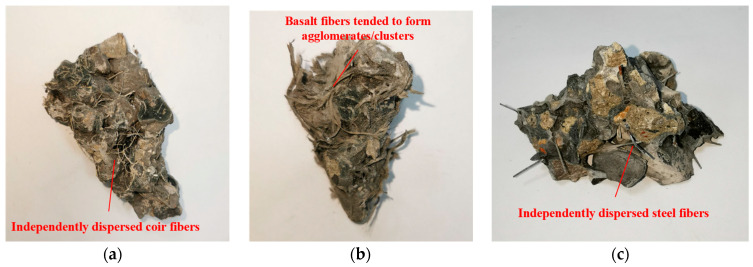
Distribution of fibers in concrete. (**a**) Coir fiber; (**b**) basalt fiber; (**c**) steel fiber.

When the fiber content is low, the added fibers provide additional restraint beyond the contact points between aggregates, effectively enlarging the inter-aggregate gaps. This phenomenon is particularly pronounced for coir fibers and steel fibers, which distribute uniformly within the GRAPC, significantly increasing the pore volume, as shown in Figure 10a,c. Meanwhile, given a constant paste content, fibers can bind a portion of the geopolymer paste, preventing its flow from clogging the pores and thereby enhancing the interconnected porosity between aggregates. However, excessive fiber content adversely affects the porosity of the concrete. On one hand, an excess of fibers tends to entangle and form clumps during mixing, hindering the formation of a homogeneous concrete mixture. On the other hand, fibers that adsorb excessive paste and interconnect with each other can form a dense fibrous network, which occupies the originally interconnected pores within the concrete, consequently reducing the effective porosity. This effect is directly observed in the agglomerated state of basalt fibers, as revealed in Figure 10b. Similar observations have been reported in hybrid fiber-reinforced concrete, where weak fiber dispersion was identified as a main reason for the deterioration of the large pore structure [31].

In summary, the regulation of porosity by fibers is a balance between two competing effects: pore creation at optimal dispersion and lower dosages, versus pore blockage due to paste absorption and fiber agglomeration at higher dosages.

#### 4.2.2. Mechanisms of Fiber Type and Content in Compressive Strength Modification

The incorporation of coir and basalt fibers at 0.5% led to a reduction in compressive strength, as shown in Figure 6a,b. For basalt fibers, two interrelated mechanisms are responsible. First, their fine diameter gives them a high specific surface area, causing them to adsorb excessive geopolymer paste during mixing. This deprives the matrix of binders needed to effectively bond the aggregates, thereby weakening the interfacial transition zones and reducing overall strength. Second, their strong tendency to agglomerate forms localized fiber bundles, further increasing the amount of geopolymer material adsorbed by the fibers, as shown in Figure 7c. For coir fibers, although they were well-dispersed and formed a uniform network within the matrix (Figure 7a,b), their low intrinsic modulus and strength limited their ability to bear or transfer stress. In summary, the strength reduction observed with coir and basalt fibers suggests that these fibers may be less suitable for strength-critical applications. However, coir fibers, with their significant contribution to porosity and ductility, are potentially useful in non-structural applications.

In contrast, Figure 6c reveals that steel fibers significantly enhanced the compressive strength. This enhancement is primarily due to their high tensile strength (≈650 MPa) and restraint of lateral deformation, which allows them to bear tensile stresses induced during compression and resist bending [32,33]. However, despite this strength gain, their relatively smooth surface and high stiffness resulted in limited interfacial adhesion, as evidenced by the sparse and uneven paste coating, as shown in Figure 7e. This also explains why further increasing the steel fiber content from 0.25% to 0.5% resulted in only marginal strength gain, with the compressive strength remaining nearly unchanged. The weak interfacial bond limits the efficiency of additional fibers in transferring load, making the strength enhancement less sensitive to fiber dosage beyond the optimal level.

The divergent compressive strength responses of the three fibers are governed by a trade-off between the fibers’ intrinsic properties and their dispersion/interfacial behavior. Steel fibers excel in load transfer but suffer from weak bonding; coir and basalt fibers are intrinsically too weak to contribute to strength enhancement.

### 4.3. Limitations and Future Work

The optimal mix proportion and the quantitative results (e.g., compressive strength values, porosity percentages) are based on specific raw materials and curing conditions. The observed trends, including that incorporating fibers improves the ductility of GRAPC regardless of fiber type, that uniform fiber dispersion is a key factor in improving effective porosity, and that steel fibers are more effective in enhancing compressive strength compared with coir and basalt fibers, are expected to extend to similar GRAPC systems. Additionally, durability performance (e.g., freeze–thaw resistance, sulfate resistance, and shrinkage) was not evaluated and should be investigated in future work.

## 5. Conclusions

This study presents a preliminary investigation into the effects of fiber type (coir, basalt, and steel) and dosage (0.25% and 0.5%) on the mechanical properties, porosity, failure mode, and microstructure of geopolymer recycled aggregate porous concrete (GRAPC). Through orthogonal experimental design, the optimal mix proportion was determined as slag content = 40%, alkali modulus = 1.4, and alkali content = 8%, which served as the baseline for subsequent fiber reinforcement studies.

The incorporation of fibers influenced the physical and mechanical properties of GRAPC in a fiber type-dependent manner. Steel fibers at 0.25% dosage enhanced compressive strength by approximately 25%, effectively bearing and transferring stress within the matrix due to their high tensile strength and restraint of lateral deformation. In contrast, 0.5% coir and basalt fibers reduced compressive strength by about 20.5% and 22.2%, respectively, with coir fibers due to their low intrinsic modulus and strength, and basalt fibers due to excessive paste adsorption and agglomeration arising from their fine diameter and high specific surface area. Regarding porosity, coir and steel fibers at 0.25% dosage increased effective porosity by 18.4% and 17.4%, respectively, by enlarging inter-aggregate gaps and preventing paste from clogging pores, owing to the uniform dispersion of the fibers. Basalt fibers showed limited porosity enhancement due to their tendency to agglomerate and form localized fiber bundles.

All three fibers promoted a more ductile-like failure mode, resulting in a “damaged yet integral” morphology compared to the brittle fracture of plain GRAPC. Coir fibers provided the greatest improvement in post-peak deformation capacity, followed by basalt and steel fibers. This order is mainly attributed to differences in fiber–matrix interfacial bonding. Coir fibers formed a well-dispersed three-dimensional network within the matrix, effectively bridging cracks and restraining aggregate displacement. Basalt fibers, despite their flexibility, exhibited agglomeration that limited the formation of a uniform bridging network. Steel fibers, while beneficial for strength, showed limited ductility improvement due to their lower hydrophilicity and weaker interfacial adhesion with the geopolymer matrix.

The findings demonstrate that fiber-reinforced GRAPC has promising potential for non-primary load-bearing engineering fields such as slope protection, municipal landscaping, and permeable pavements. By combining geopolymer, recycled aggregates, and fibers in a porous concrete framework, the material achieves both environmental friendliness and improved mechanical performance, mitigating the limitations of low strength and brittleness typically associated with such systems. As a preliminary study, this work establishes a foundational understanding of fiber selection and mix proportion optimization for GRAPC. The ability to tailor porosity, strength, and ductility through fiber selection offers a versatile approach for designing sustainable infrastructure materials. Future research could focus on durability performance (e.g., freeze–thaw resistance, sulfate resistance, drying shrinkage) to further optimize fiber-reinforced GRAPC.

## Figures and Tables

**Table 1:** Chemical composition of ground granulated blast furnace slag (GGBS) and fly ash (FA).

Material	CaO	SiO_2_	Al_2_O_3_	SO_3_	Fe_2_O_3_	MgO
GGBS	34.0	34.5	17.7	1.6	1.0	6.0
FA	5.6	43.0	23.0	0.8	2.5	1.0

**Table 2:** Basic properties of recycled aggregate.

Material	Bulk Density /(kg/m^3^)	Apparent Density /(kg/m^3^)	Void Content /%	Water Absorption /%	Crushing Index /%
recycled aggregate	1418	2653	46.6	3.8	12.8

**Table 3:** Mix proportions of the orthogonal experimental design (kg/m^3^).

No.	GGBS	FA	NaOH	Na_2_SiO_3_	Recycled Aggregate	Water
1	106.3	159.45	15.69	33.81	1418	119.74
2	97.0	145.50	18.83	53.89	1418	119.74
3	99.4	149.10	13.76	52.71	1418	119.74
4	147.8	98.53	21.82	47.01	1418	119.74
5	151.2	100.80	16.31	46.66	1418	119.74
6	155.8	103.87	11.50	44.06	1418	119.74
7	204.5	51.13	18.87	40.65	1418	119.74
8	210.0	52.50	13.59	38.89	1418	119.74
9	190.8	47.70	15.85	60.70	1418	119.74

**Table 4:** Mix proportions of fiber-reinforced specimens (kg/m^3^).

Mix ID	GGBS	FA	NaOH	Na_2_SiO_3_	Recycled Aggregate	Water	Fiber
Without fibers	105	157.5	13.59	38.9	1418	119.7	0
Coir—0.25%	105	157.5	13.59	38.9	1418	119.7	6.1
Coir—0.5%	105	157.5	13.59	38.9	1418	119.7	12.2
Basalt—0.25%	105	157.5	13.59	38.9	1418	119.7	13.5
Basalt—0.5%	105	157.5	13.59	38.9	1418	119.7	27.0
Steel—0.25%	105	157.5	13.59	38.9	1418	119.7	37.5
Steel—0.5%	105	157.5	13.59	38.9	1418	119.7	75.0

## Data Availability

The raw data supporting the conclusions of this article will be made available by the authors on request.

## Abbreviations

The following abbreviations are used in this manuscript:

GRAPC	Geopolymer Recycled Aggregate Porous Concrete
SEM	Scanning Electron Microscopy
GGBS	Ground Granulated Blast Furnace Slag
FA	Fly Ash
SD	Standard Deviation
